# DNA recovery and analysis from skeletal material in modern forensic contexts

**DOI:** 10.1080/20961790.2018.1515594

**Published:** 2018-10-08

**Authors:** Krista E. Latham, Jessica J. Miller

**Affiliations:** Biology Department, Human Identification Center, University of Indianapolis, Indianapolis, IN, USA

**Keywords:** Forensic sciences, forensic anthropology, forensic genetics, individual identification, molecular taphonomy, skeletal DNA Extraction, DNA profiles, DNA databases

## Abstract

The generation of a DNA profile from skeletal remains is an important part of the identification process in both mass disaster and unidentified person cases. Since bones and teeth are often the only biological materials remaining after exposure to environmental conditions, intense heat, certain traumatic events and in cases where a significant amount of time has passed since the death of the individual, the ability to purify large quantities of informative DNA from these hard tissues would be beneficial. Since sampling the hard tissues for genetic analysis is a destructive process, it is important to understand those environmental and intrinsic factors that contribute to DNA preservation. This will serve as a brief introduction to these topics, since skeletal sampling strategies and molecular taphonomy have been discussed in depth elsewhere. Additionally advances in skeletal DNA extraction and analysis will be discussed. Currently there is great variation in the DNA isolation methods used by laboratories to purify DNA from the hard tissues; however, a standardized set of short tandem repeat (STR) loci is analyzed by many US laboratories to allow for comparisons across samples and jurisdictions. Recent advances have allowed for the generation of DNA profiles from smaller quantities of template DNA and have expanded the number of loci analyzed for greater discriminatory power and predictions regarding the geographic ancestry and phenotype of the individual. Finally, utilizing databases and expanding the number of comparison samples will be discussed in light of their role in the identification process.

## Introduction

The field of forensic science is constantly changing as new methods and techniques become available and validated for use in the medicolegal system. The field of forensic genetics, which applies genetic science to issues of identification and criminal investigation, has experienced an especially rapid rate of advancement during the last decade. From expanding the number of loci commonly utilized in forensic investigations, to the growth of DNA databases, to the increased sensitivities of DNA technologies, genetic evidence has grown to play an integral role in the criminal justice community [1–5].

DNA obtained from evidentiary items may be valuable in placing a person at the scene of a crime or in contact with an object associated with a criminal act. DNA data may also be an important piece of evidence in exonerating an innocent individual. Additionally, the generation of DNA profiles from human remains can be essential in the personal identification of missing persons and mass disaster victims. All of these genetic investigations utilize the same underlying approach: the generation of DNA profiles from the unknown evidentiary sample and a known reference sample, followed by a comparison. If the DNA profiles are not consistent then a person can be eliminated from contributing their transfer DNA to an item or the identification hypothesis must be rejected. If the DNA profiles are consistent, statistical calculations are performed to provide information regarding the confidence in the match [5,6].

The generation of a DNA profile from bones and teeth is an important part of the identification process in both mass disaster and unidentified remains cases. Skeletal elements are often the only biological materials remaining after exposure to environmental conditions, intense heat, certain traumatic events, and in cases where a significant amount of time has passed since the death of the individual. Therefore, the ability to purify large quantities of informative DNA from the hard tissues would be beneficial in numerous forensic and archeological contexts.

## Molecular taphonomy

The ability to generate DNA profiles from human remains is often challenging as the DNA begins to degrade immediately after the cells die. Cell death by autolysis occurs once a cell is no longer in contact with the body’s circulating oxygen supply. This results in the rupture of the cellular membranes and a release of enzymes that will begin to degrade cellular structures. The DNA begins to degrade as soon as it comes in direct contact with the enzymes, since the chemical reactions that subsequently occur will act to modify and break the DNA strands [7,8]. After the initial onslaught by endogenous enzymes, the DNA is further degraded by microbes present both within and external to the body. DNA degradation continues over long periods of time as hydrolytic and oxidative chemical reactions damage the molecules by fragmentation and chemical modification [7–9].

Analyzable DNA often persists in bones and teeth much longer than in the soft tissues of the body, because the rigid structure of bones and teeth provide some protection against DNA degradation. The DNA molecules become chemically bound to the hydroxyapatite of the hard tissues, which stabilizes the DNA and provides some protection against degradation. Therefore, the extent of skeletal DNA degradation is, in part, related to gross bone and gross tooth degradation [7–10]. A variety of factors act to accelerate or slow the biological degradation process. Molecular taphonomy is the study of the various intrinsic and extrinsic factors that influence degradation of the body’s molecular structures, such as DNA [7].

A variety of environmental factors can act to create differential preservation in different skeletons, in different bones within the same skeleton, and even variations in DNA quality and yield across the same bone. Therefore, the depositional environment plays a greater role in contributing to molecules degrading at different rates than the absolute age of the DNA sample [11–16]. Additionally, intrinsic factors like bone type and density can contribute to favourable DNA preservation. None of these factors operates in isolation, and the combination of specific variables influencing a particular set of skeletonized remains may work together or in opposition regarding molecular degradation. Furthermore, skeletal DNA degradation is influenced by the rate and level of bone degradation. Since sampling the hard tissues for genetic analysis is a destructive process, it is crucial to understand those factors that are most promising for DNA preservation [7]. The following sections briefly address some of these factors; however, it is important to note that while each factor is being discussed in isolation, the intricacies of a particular death scene may create a complex web of interactions between these variables.

### Temperature

The degradation of biological material occurs as the result of a series of chemical reactions, with autolysis displaying a maximum chemical activity at 34 °C–40 °C. All chemical reactions are heavily influenced by temperature, with a twofold to threefold increase in the reaction rate for every 10 °C increase in temperature [7,10,17,18]. Additionally, temperature influences the microbial activity associated with biological decomposition, with warmer temperatures promoting microbial growth. Thus, cooler temperatures will favour DNA preservation in general. However, research suggests that there are some circumstances in which mild heating may be beneficial in order to increase DNA yields from hard tissues because it makes the bone brittle and better able to release the DNA during the purification process [7,19,20].

### Moisture levels

The presence of moisture in the depositional environment can impact biological decomposition. Water molecules participate in hydrolytic reactions that act to fragment and modify DNA molecules. Generally, the more groundwater or humidity present in the depositional environment, the greater the likelihood of DNA damage. However, there are situations in which particular water environments may influence how the other environmental variables affect molecular preservation. For example, burial in a peat bog may actually be beneficial to DNA preservation because it creates a low oxygen environment and burial in salt water could slow DNA degradation by reducing the levels of microbial activity. On the other hand, groundwater contributes to bone degradation, which in turn contributes to DNA loss [7,21,22].

### Oxygen levels

Oxygen molecules participate in oxidative reactions that modify DNA bases and create lesions in the DNA strands. This process leads to further degradation as well as a helical distortion that can complicate later genetic analyses [7,18,23]. Additionally, oxygen levels influence the rate and extent of microbial decomposition. Therefore, oxygen rich environments will lead to greater DNA degradation.

### Microorganism activity

Both endogenous and exogenous microorganisms contribute to biological decomposition. The microbes do not digest the DNA directly, but rather they digest the protein component of bone making the skeletal DNA more prone to damage. In addition, microorganisms that participate in biological decomposition produce enzymes that fragment DNA molecules [7,9,17]. Many of the environmental factors discussed in this section can act to facilitate or impede bacterial access to the remains and activity levels. In general, greater microbial access results in greater DNA degradation.

### Soil composition

The chemical composition of the soil can complicate later genetic analyses conducted on skeletal DNA molecules. Bones and teeth reach a chemical equilibrium with the depositional environment via mineral leaching and the uptake of different solutes from the soil. This process can lead to bone degradation and chemical changes of the hydroxyapatite, both factors that can impact the rate and degree of DNA degradation. Additionally, soil solutes such tannins and humic acids may co-purify with the DNA during the extraction process and inhibit certain subsequent genetic analyses [7,24–26].

### pH

Biological decomposition occurs more rapidly in acidic and alkaline (rather than neutral) environments. Chemical modifications to hydroxyapatite and DNA are influenced by the pH of the depositional environment. The rate of microbial decomposition is also influenced by the pH of the depositional environment [7,25,27,28]. Thus, DNA is less prone to damage in neutral or near neutral environments.

### Bone type

In addition to the many environmental factors that affect DNA degradation, intrinsic factors like bone type can play a role in the process of DNA decomposition as well. Bone size and construction can impact skeletal DNA preservation. Larger bones tend to survive better and are therefore differentially available for sampling for genetic analysis. The dense cortical portions of lower limb bones and the harder tissues of teeth tend to be consistently reliable in generating DNA profiles compared to less dense spongy bone [7,15,29–32]. Therefore, an understanding of the skeletal elements most likely to produce a DNA profile should be considered rather than sampling based merely on convenience.

## Skeletal DNA extraction

Purifying DNA from bones and teeth often requires modification of the DNA extraction techniques utilized for other types of biological samples. However, there is great variation in the DNA isolation methods used by laboratories to purify DNA from hard tissues. Many of these processes begin with a step aimed at removing contaminating DNA transferred to the surface of the bone or tooth that would contribute to the generation of a mixed DNA sample. Decontamination can be accomplished by physically removing the outer bone surface, by immersing the bone or tooth in a bleach solution, or by exposure to ultraviolet radiation [4,7,33]. After surface decontamination, the hard tissues are often pulverized and subsequently incubated in extraction buffer and proteinase K, which together dissolve the organic and inorganic portions of the bone tissue. The amount of bone powder used in this step varies greatly from laboratory to laboratory with most published protocols calling for as high as 2.5 g to as little as 0.2 g of starting material. Grinding the sample into a powder exposes a greater surface area to the various chemicals employed in the DNA extraction process, therefore releasing a greater amount of DNA from the hydroxyapatite mineral matrix. The DNA is then purified from the other dissolved materials utilizing a variety of techniques including commercial kits and organic solvents [4,7,33–40].

The goal of skeletal DNA extraction techniques is to maximize DNA yield, minimize any additional DNA damage and remove any inhibitors that may co-purify with the skeletal DNA and interfere with later genetic analyses. While the physical robustness of bones and teeth plays a role in DNA preservation, the added steps to purify the DNA from the hard tissues may further damage the DNA. Several studies [7,19,20,30,41,42] have investigated whether subjecting bone to mild heating may increase DNA yields. These studies suggest that mild heating makes the hard tissues brittle and better able to release the DNA during the purification process and reduces the amount of moisture in the bone, thus slowing damage due to hydrolytic reactions. Reidy et al. [20] were able to obtain analyzable DNA from a previously unsuccessful bone sample after subjecting the bone to 100 °C for 72 h. Madonna et al. [41] systematically tested the influence of temperature on DNA quantity and quality. They found that heating bone at 90 °C for up to 72 h increased DNA yield in their study samples. However, there is a point at which temperatures peak and compromise the integrity of the DNA molecules. Maciejewska et al. [43] investigated the ability to generate DNA profiles from soft and hard tissues exposed to high temperatures for short periods of time. They were able to generate full DNA profiles from soft tissues exposed to 900 °C for 5 min but had limited success with hard tissues. However, Zgonjanin et al. [44] report modifications to their DNA extraction protocol that have allowed them to successfully generate DNA profiles from burned bodies. Since heat is generally viewed as accelerating DNA damage, other studies have looked at removing all steps that generate heat from the skeletal DNA purification process by making modifications to the grinding process. Some laboratories have incorporated liquid nitrogen into their grinding procedure and others have utilized freezer mills [45–48]. Currently there is not an agreement in the forensic DNA community as to whether the benefit of subjecting bones to heat to render them brittle to potentially increase DNA yield outweighs the potential damage the heat causes to the DNA molecules.

Additionally, advances have been made in extraction techniques and amplification kits to reduce the amount of biological material destroyed during the DNA isolation procedure. Optimizing the DNA extraction process and increased sensitivity of DNA kits has allowed laboratories to slowly decrease the amount of starting material needed for the extraction process, allowing for minimal destruction of the skeletal materials [39,40,46,48,49]. Many studies [15,29–32] have investigated the relationship between skeletal element (bone type) and DNA yield as a way to sample skeletal remains based on the likelihood of generating an informative DNA profile. They suggest that compact bone tends to yield greater amounts of DNA than spongy bone. In addition, these studies found differences in the success rates of genetic analyses between the compact bone of the limbs with the upper limbs being successful less than 50% of the time and the lower limbs being more effective.

Another factor contributing to the differential success of genetic analyses is the presence of polymerase chain reaction (PCR) inhibitors that co-purify with the extracted skeletal DNA. Most techniques currently employed by forensic DNA laboratories in the US begin by making many copies of the DNA areas of interest analyzed for identification purposes. The process of copying the DNA is called PCR. The PCR process relies on enzymes and temperature manipulation to produce millions of copies of the target DNA. PCR is able to detect and copy the DNA from as little as a single cell, which is advantageous when working with skeletonized human remains [5,50,51]. However, the presence of solutes from the depositional environment and later co-purified with the skeletal DNA can serve as PCR-inhibitors by blocking the enzymatic reactions that occur during the PCR process. To overcome the effect of PCR inhibitors, one can employ techniques that remove inhibitors prior to, during, or after DNA extraction, or that suppress the impact of inhibitors during the PCR process. Some commercial DNA extraction kits remove or reduce the amount of inhibitors [52–54].

## DNA analysis

Genetic analysis is a fundamental tool in the positive identification of skeletonized remains. However, choosing the appropriate genetic test depends upon the specific question that needs to be addressed, the ability to obtain a reference sample, and the condition of the DNA sample. Human cells contain two types of DNA: nuclear and mitochondrial. Portions of the nuclear genome are most often utilized during forensic investigations because the extreme amount of variation from person to person increases the probability that the DNA profile will be unique and individualizing.

### Nuclear DNA analysis

The nuclear genome is comprised of approximately six billion base pairs per somatic (body) cell. The nuclear DNA is packaged into chromosomes and located inside the cell nucleus. There are 46 chromosomes found inside human somatic cells, with 23 being inherited from the individual’s mother and 23 being inherited from the individual’s father. Therefore, a person’s nuclear DNA is a unique representation of all their ancestor’s DNA and in most cases (except identical twins) is individualizing.

Forensic analyses in the US utilize a standardized set of autosomal short tandem repeat (STR) loci. STRs are noncoding DNA sequences consisting of tandem repeats of a core repeating unit approximately 2–6 nucleotides long. STRs are highly polymorphic in that the number of times the core is repeated varies from person to person. A standardized set of 13 STRs were chosen to form the basis for US forensic DNA profiling in 1997 during a large scale FBI sponsored initiative. These loci form the foundation of the Combined DNA Index System (CODIS) national database, which was launched in 1998. The 13 CODIS core STR loci are located on autosomal chromosome numbers 2, 3, 4, 5, 7, 8, 11, 12, 13, 16, 18 and 21. The standardization of the loci used in the generation of a DNA profile allows for the direct comparison of DNA profile results between various laboratories and law enforcement agencies in the US [6].

A number of commercial kits from several vendors are available for multiplex PCR amplification of the STRs used to generate a DNA profile. While the 13 core STR loci have the ability to produce random match probabilities at rarer than one in a trillion in unrelated individuals in the population at large, many companies are increasing the number of loci being tested in their kits. For example, the AmpFℓSTR® Identifiler® Plus PCR Amplification Kit amplifies 15 STR loci, the Promega PowerPlex® 16 HS System amplifies 16 STR loci, and the Applied Biosystems™ GlobalFiler™ PCR Amplification Kit amplifies 21 STR loci. The additional STR loci have the ability to push random match probabilities into ranges that many people cannot comprehend, such as rarer than one in a nonillion in unrelated individuals in the population at large [5,6].

Several advances in DNA typing technology have addressed the issue of generating DNA profiles from degraded samples. In addition to increasing the discriminatory power of DNA evidence, the newer commercial kits are also more sensitive in that they can produce a full DNA profile from smaller amounts of template DNA. Validation studies demonstrate that full DNA profiles can be generated from as little as 125 pg of DNA, which corresponds to only 15–20 human cells [3,5,6,55,56]. Additionally, kits have been developed to produce amplicons for the commonly used STR markers that are reduced in size when compared to the commonly available commercial kits. The amplicon size is reduced to less than 300 base pairs (bp) by moving the primers as close as possible to the STR targets, but this does not change the loci utilized in DNA profile generation [57,58].

Employing additional nuclear loci can prove useful for challenging samples and can assist in predictions regarding the ancestry and phenotype of the individual in question. In some cases involving degraded DNA, even the additional step of reducing the STR amplicon size will not produce a DNA profile. In such situations, single nucleotide polymorphisms (SNPs) with amplicon sizes ranging from only 60–80 bp may be an alternative. In human identification cases from skeletonized remains or in mass disaster situations, the DNA may be severely fragmented and SNPs could provide more genetic results than STRs. However, SNPs are not as polymorphic as STRs and SNP profiles will not be directly comparable to the profiles generated utilizing the common STR loci [59]. Additionally, SNPs have been identified that provide some information regarding the geographic ancestry of the individual. These markers are often called ancestry informative markers (AIMs). Currently, AIM panels have been able to distinguish among major continental groups and may be utilized for ancestry inference as an investigative tool. However, it is important to note that broad continental ancestry predictions utilizing these genetic tools may not translate simply to social race categories. Furthermore, misclassifications can occur if the global ancestry of the person in question is not represented in the reference populations or if the individual has a significant amount of genetic admixture [59–62]. Other SNPs are being analyzed to provide information regarding the physical appearance of the person in a process called DNA phenotyping. This can be used to provide investigative leads regarding suspects and in missing persons cases. Skin, eye and hair pigmentation, as well as several other externally visible characteristics like stature can currently be predicted using various SNP panels [59,63,64]. However, large numbers of SNPs and AIMs must be analyzed in order to provide individualizing data, to estimate geographic ancestry or predict phenotypic characteristics.

Clearly the analysis of nuclear DNA can provide valuable information regarding the identification of an individual. Analyzing at least 13 STR loci can provide positive identification for missing persons and victims of mass disasters that may not be able to be identified using other traditional means, such as fingerprints, in situations when direct reference samples are available for comparison. The analysis of additional SNP loci may also provide information regarding the person’s geographic ancestry and physical appearance. However, the ability to obtain and amplify nuclear DNA is not possible in all situations and in some cases the mitochondrial genome may be needed during forensic investigations.

### mtDNA analysis

The mtDNA is located outside of the cell nucleus in cellular structures called mitochondria. While each nucleus contains only one copy of each of the 46 human chromosomes, the mitochondria contain multiple identical copies of the mitochondrial genome. This high copy number makes the mtDNA a favourable target for degraded samples since the probability of obtaining analyzable mtDNA is greater than for nuclear DNA. However, the uniparental inheritance pattern of the mitochondrial genome means that an individual’s mtDNA will be the same as all of their consanguineous maternal relatives. Therefore, mtDNA profiles can only provide a circumstantial identification. However, mtDNA profiles are still important in forensic investigations when samples are degraded and do not produce nuclear profiles, when supplemental genetic information is needed or when suitable autosomal STR references are not available [4,7,9].

While a standardized set of STRs is analyzed in nuclear DNA analysis, mtDNA variation is usually determined by directly sequencing a portion of the mitochondrial genome referred to as the hypervariable region. Because recombination does not occur in mtDNA, this same sequence will be inherited through the maternal lineage of a family. Therefore, the mtDNA sequence of a close maternal relative can be compared to that of an unidentified person. If the sequences are interpreted as not being consistent, the individual can be excluded as belonging to that genetic line. In addition to providing information regarding the identity of an individual, new research suggests that mtDNA can also be utilized to estimate a person’s age at death. Relationships have been detected between the accumulation of mtDNA mutations and aging in different tissues. The relationship is likely due to the accumulation over time of free radicals within the mitochondria that leads to oxidative damage of the mtDNA. The ability to estimate age at death based on mtDNA damage in bones and teeth could provide investigative leads in missing persons and mass disaster cases [65–67].

### Y-Chromosome analysis

The Y-chromosome is part of the nuclear genome, yet its inheritance pattern allows it to be employed in genetic analyses in a similar fashion as mtDNA. While mtDNA follows a family’s unbroken female line, the Y-chromosome follows a family’s unbroken male line. A majority of the Y-chromosome does not recombine with its corresponding X-chromosome and is referred to as the non-recombining portion of the Y-chromosome (NRY). PCR amplification of STRs located within the NRY can be useful in male specific-identifications [6,7,62].

The significance of Y-chromosome analysis in a forensic context relies on males carrying a copy of the Y-chromosome while females do not. Genetic tests designed to only examine the male DNA in a sample can be extremely valuable in criminal cases involving male perpetrators, such as sexual assault cases. In 2003, the US Scientific Working Group on DNA Analysis Methods (SWGDAM) recommended a core set of Y-STRs to be analyzed in forensic investigations. Commercial kits, such as the Promega PowerPlex® Y and Applied Biosystems^TM^ Yfiler, target these loci in addition to the European core Y-STR loci [6].

## Databases

DNA profiles generated from standardized nuclear and mtDNA analyses are used in US DNA databases. The power of DNA databases lies in the ability to compare DNA profiles between reference, crime scene and unidentified person samples across multiple jurisdictions. The rise of such databases has proven vital to the forensic community and expanding DNA collection laws have provided a growing number of comparison samples. Over 190 public crime labs in the US are interconnected via CODIS. This system is managed and distributed through the Federal Bureau of Investigation (FBI). DNA profiles are entered into different indexes (unidentified human remains, missing person, relatives of missing person, offender, arrestee, detainee and forensic) and then compared to determine potential matches. Additionally, this system is organized into Local DNA Index System (LDIS), State DNA Index System (SDIS) and National DNA Index System (NDIS) levels [2,5].

Another database that has proven to be a valuable tool to the forensic community is The National Missing and Unidentified Person System (NamUs) which contains both genetic and non-genetic data regarding missing persons, unidentified decedents and unclaimed individuals. The power of NamUs lies in the ability to consolidate all the data, documents and images surrounding the case in one place. Users have differential access depending on their role in the investigation, with some information being available even to the general public. This system is managed by the University of North Texas (UNT) Health Science Center through a cooperative agreement with the National Institute of Justice (NIJ). While this system does not actively compare the DNA data between the missing and unidentified individuals, it does serve as a place to store that information. Additionally, through funding provided by the NIJ, the UNT Health Science Center will process the DNA samples obtained from unidentified individuals at no charge to the submitting agency [2,68].

## Future considerations

The field of forensic DNA will continue to advance in ways that will positively impact the ability to identify skeletonized remains and the victims of mass disasters. Advancements will continue to lower the DNA detection limits and optimize the PCR amplification of shorter polymorphic loci to allow for DNA profiles to be generated from samples that would previously be unsuccessful. Adopting next-generation sequencing (NGS) or massive parallel sequencing (MPS) will allow for the analysis of multiple STRs and SNPs, as well as whole mitochondrial sequencing from evidentiary samples. Additionally, advancements in automated instrumentation will continue to reduce the amount of time it takes to generate a DNA profile. Continuing to expand the number of comparison samples in DNA databases will ultimately lead to more identifications. Improving the ability to interpret DNA results from challenging samples will be an important aspect of the future of the field. Finding ways to reduce the cost of genetic analyses will reduce the backlogs and allow for more samples to be processed. Finally, adequate training and funding must be provided in order to recognize that sound research based upon the scientific method is the key to advancement in any forensic science field.
